# Using pre-fracture mobility to augment prediction of post-operative outcomes in hip fracture

**DOI:** 10.1007/s41999-023-00767-0

**Published:** 2023-03-31

**Authors:** Thomas A. Stubbs, William J. Doherty, Andrew Chaplin, Sarah Langford, Mike R. Reed, Avan A. Sayer, Miles D. Witham, Antony K. Sorial

**Affiliations:** 1 AGE Research Group, NIHR Newcastle Biomedical Research Centre, Translational and Clinical Research Institute, Faculty of Medical Sciences, Newcastle University and Newcastle Upon Tyne Hospitals NHS Foundation Trust, Campus for Ageing and Vitality, Newcastle-Upon-Tyne, NE4 5PL UK; 2 Department of Trauma and Orthopaedics, Northumbria Healthcare NHS Foundation Trust, Newcastle Upon Tyne, NE27 0QJ UK; 3 Institute for Cell and Molecular Biosciences, Newcastle University, International Centre for Life, Newcastle Upon Tyne, NE1 3BZ UK

**Keywords:** Frailty, Mobility, Hip fracture, Nottingham Hip Fracture Score, Prognostication

## Abstract

**Purpose:**

Pre-operative scores based on patient characteristics are commonly used to predict hip fracture outcomes. Mobility, an indicator of pre-operative function, has been neglected as a potential predictor. We assessed the ability of pre-fracture mobility to predict post-operative outcomes following hip fracture.

**Methods:**

We analysed prospectively collected data from hip fracture surgery patients at a large-volume trauma unit. Mobility was classified into four groups. Post-operative outcomes studied were mortality and residence at 30 days, medical complications within 30- or 60-days post-operatively, and prolonged length of stay (LOS, ≥ 28 days). We performed multivariate regression analyses adjusting for age and sex to assess the discriminative ability of the Nottingham Hip Fracture Score (NHFS), with and without mobility, for predicting outcomes using the area under the receiver operating characteristic curve (AUROC).

**Results:**

1919 patients were included, mean age 82.6 (SD 8.2); 1357 (70.7%) were women. Multivariate analysis demonstrated patients with worse mobility had a 1.7–5.5-fold higher 30-day mortality (*p* ≤ 0.001), and 1.9–3.2-fold higher likelihood of prolonged LOS (*p* ≤ 0.001). Worse mobility was associated with a 2.3–3.8-fold higher likelihood of living in a care home at 30-days post-operatively (*p* < 0.001) and a 1.3–2.0-fold higher likelihood of complications within 30 days (*p* ≤ 0.001). Addition of mobility improved NHFS discrimination for discharge location, AUROC NHFS 0.755 [0.733–0.777] to NHFS + mobility 0.808 [0.789–0.828], and LOS, AUROC NHFS 0.584 [0.557–0.611] to NHFS + mobility 0.616 [0.590–0.643].

**Conclusion:**

Incorporating mobility assessment into risk scores may improve casemix adjustment, prognostication following hip fracture, and identify high-risk patient groups requiring enhanced post-operative care at admission.

**Supplementary Information:**

The online version contains supplementary material available at 10.1007/s41999-023-00767-0.

## Key summary points

**Aim:**

To study whether pre-fracture mobility be used to strengthen post-operative outcome prediction following hip fracture.

**Findings:**

Patients with better mobility had significantly improved outcomes and the mobility variable was able to independently predict outcomes while enhancing risk prediction when combined with the Nottingham Hip Fracture Score. Incorporating mobility assessment into risk scores may improve casemix adjustment, prognostication following hip fracture, and identify high-risk patient groups requiring enhanced post-operative care at admission.

**Message:**

Mobility information available at admission could facilitate prognostication, discharge planning, bed management and risk aversion, as well as informing discussions between clinical teams and patients about post-operative recovery.

**Supplementary Information:**

The online version contains supplementary material available at 10.1007/s41999-023-00767-0.

## Introduction

In 2020, there were over 63,000 admissions to hospital for hip fractures in the UK [1]. Hip fracture remains a common serious injury in the older UK population and carries a high rate of morbidity and mortality [1]. The 30-day mortality rate following hip fracture surgery in 2019 was 6.5%, increasing to 8.3% in 2020 [1, 2]. Hip fractures are estimated to cost the NHS £1.1 billion annually [3], with much of the cost of care attributable to post-operative length of stay (LOS) [4]. In the UK, the mean acute LOS was 15.2 days in 2020 [1].

Predicting outcomes after hip fracture is important for identifying high-risk patients who may benefit from additional care, and for adjusting for casemix between different centres. Existing tools, such as the Nottingham Hip Fracture Score (NHFS), demonstrate moderate discriminant ability for post-operative mortality but are less accurate in predicting postoperative complications and length of stay [5–7]. Such scores do not currently include measures of mobility prior to hip fracture.

Mobility is closely related to physical frailty, an important health state that is a predictor of a range of adverse outcomes following hip and acetabular fractures, including death, prolonged hospital stay, falls and the need for nursing home care [6–9]. Mobility is directly related to frailty; the ability to mobilise relies on appropriate function of multiple body systems, including the musculoskeletal system, cardiorespiratory system, and both the central and peripheral nervous systems. A national audit, the National Hip Fracture Database (NHFD), routinely collects data on mobility prior to hip fracture and following surgery. These data could provide insights into a measure related to frailty, and potentially improve hip fracture prognostication.

Little work has been done to demonstrate whether mobility can improve the predictive performance of validated measures such as the NHFS. The aim of this analysis was to assess the effect of pre-fracture mobility on post-operative outcomes including LOS, post-operative residence and post-operative complications, and to test whether combining mobility with the NHFS improved its discriminant ability for these outcomes.

We hypothesised that patients with better mobility would have lower mortality, shorter LOS, be more likely to be discharged back to their own homes or residences with a higher level of independence and suffer fewer complications.

## Methods

### Data source

Data were collected prospectively on hip fracture patients attending Northumbria Healthcare NHS Foundation Trust as part of NHFD data collection between 1st April 2014 and 31st December 2018. In-patient data were collected during admission and entered into a local database by trained specialist nurses, prior to upload to the NHFD [2]. NHFD data were exported and merged with an export of Hospital Episode Statistics (HES) prior to anonymisation for analysis. All data were managed in accordance with Caldicott principles and the analysis did not require evaluation by an ethics committee, as the analyses did not require new patient contact or data collection [10]. The sources and types of data collected are summarised in Supplementary Table 1.

### Measure of pre-fracture mobility

Patients were classified into pre-defined mobility groups based on the NHFD. Mobility was classified in descending order as: Mobile outdoors without aids; Mobile outdoors with aids; Mobile indoors with aids (but does not go outside without help); Not mobile (confined to a wheelchair or bed). For some analyses, mobility was dichotomised as being either “Mobile outdoors without aids” (the best mobility state) or “Not mobile without aids”, which combined the latter three groups.

### Post-operative outcomes

Outcome variables used in the analyses were mortality and residence at 30-days post-operatively, LOS (in days) and post-operative medical complications. Residences were categorised in descending order as “own home or sheltered housing”, “residential care”, “nursing care”, “rehabilitation unit”, “hospital” or “deceased”. Patients who died within 30 days of surgery were excluded from the post-operative location analysis. A further analysis on post-operative mobility including patients who died within 30 days was completed.

Medical complications included in the analysis are listed in Supplementary Table 2, and were defined using ICD-10 or OPCS-4 codes [11, 12].

Post-operative medical complications variables were combined for use in the regression analyses. Venous thromboembolism (VTE) events were defined as pulmonary embolism (PE) or Deep Vein Thrombosis (DVT) within 60 days of surgery. Arterial thromboembolic events were defined as stroke or myocardial infarction (MI). Renal complications were defined as acute kidney injury or urinary retention. Infection was defined as pneumonia or urinary tract infection (UTI). ‘Any complication’ was an aggregate variable including all complications within 30 days post-surgery but excluding VTE events. VTE events were omitted from this variable as they were measured within 60 days of surgery.

Control variables of age and sex were also used in the analysis to account for patient differences in the mobility groups.

### Exclusions

Patients were excluded if they had missing data on their pre-fracture mobility, outcomes, age sex, or if their fracture was not operatively managed. For LOS and post-operative location analyses, patients were excluded if they died within 30 days of surgery.

The univariate and multivariate regression analysis of patient location at 30-days post-operatively included only patients who resided in their own homes prior to hip fracture, as patients admitted from residential or nursing homes were highly unlikely to be discharged to their own homes or sheltered housing following hip fracture.

Two analyses for location at 30-days post-operation were completed, the first excluded patients who died within 30 days of surgery, the second included these patients using a composite endpoint of being alive and at home at 30 days following surgery.

### Statistical analysis

Descriptive statistics were produced for each mobility group. The assessed variables were compared between the different mobility groups and significance assessed using the one-way ANOVA for parametric continuous data. Categorical data were analysed using Pearson’s Chi-squared test, or Fisher’s exact test when the minimum number of observations were less than 5.

Univariate multinomial logistic regression analysis was performed for each of the variables to create an odds ratio (OR) with a 95% confidence interval (CI) for each category of pre-fracture mobility. The OR were calculated in comparison to the referent group, which was “mobile outdoors without aids”. The analysis was then repeated as a multivariable regression with forced entry to adjust for the effects of other baseline variables.

The additional discriminant value of pre-fracture mobility was tested by generating predicted probabilities for the NHFS and the NHFS with pre-fracture mobility in binary logistic regression analyses. These predicted probabilities were then used to generating Receiver Operator Characteristic (ROC) curves and calculate *c*-statistics (with 95% CI) with respect to 30-day mortality, post-operative complications within 30 days, returning to own home within 30 days and LOS ≥ 28 days, and compared to *c*-statistics calculated for NHFS alone.

Data were analysed using SPSS v25 (IBM, New York, USA). A two-sided *p*-value of < 0.05 was taken as significant for all analyses.

## Results

**Fig. 1:**
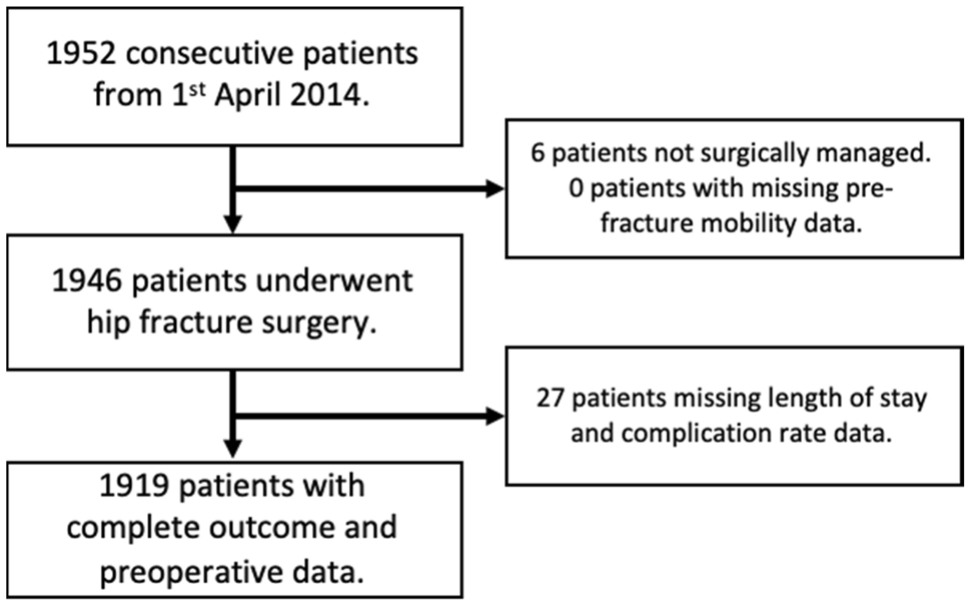
Inclusion of patients

1919 patients who were admitted between 1st April 2014 and 31st December 2018 were included in the analysis (Fig. 1). The mean age was 82.6 years (SD 8.2) and 1357/1919 (70.7%) were women. 127/1919 (6.6%) of patients died within 30 days post-operatively and 825/1919 (43.0%) were in their own home or sheltered housing at 30 days post-operatively. The median LOS for the cohort was 18 (IQR 10–30) days. Prior to hip fracture, 550 (28.7%) patients were mobile without aids, 565 (29.4%) were mobile outdoors with aids, 743 (38.7%) were mobile indoors with aids, and 61 (3.2%) were non-mobile. Baseline characteristics of patients are shown in Table 1. Characteristics of patients who were admitted from their own home or sheltered housing are available in Supplementary Table 3.

**Table 1:** Baseline characteristics of all included patients (*n* = 1919)

Variables	All patients (*n* = 1919)
Age	
Mean (SD)	82.6 (8.2)
Median (IQR)	83 (78–88)
Female sex, *n* (%)	1357 (70.7)
Pre-fracture mobility, *n* (%)	
Mobile without aids	550 (28.7)
Mobile outdoors with aids	565 (29.4)
Mobile indoors with aids	743 (38.7)
Not mobile	61 (3.2)
Pre-fracture location, *n* (%)	
Own home/sheltered housing	1413 (73.6)
Residential care	381 (19.9)
Nursing care	78 (4.1)
Hospital	47 (2.4)
LOS	
Mean (SD)	24.1 (21.5)
Median (IQR)	18 (10–30)
NHFS	
Mean (SD)	5.0 (1.6)
Median (IQR)	5 (4–6)
ASA	
Mean (SD)	2.9 (0.7)

### Mortality

In the univariate analysis, the OR for 30-day mortality was 3.4 (95% CI 2.0–6.1, *p* < 0.001) for patients requiring mobility aids, compared with those who did not.

When adjusting for age and sex, the 30-day mortality OR was 2.3 (95% CI 1.2–4.4, *p* = 0.01) for patients mobile outdoors with aids, 3.5 (95% CI 1.9–6.4, *p* < 0.001) for patients mobile indoors with aids and 5.7 (95% CI 2.3–14.2, *p* < 0.001) for patients non-mobile prior to fracture, compared to the referent group.

### Length of stay

**Fig. 2:**
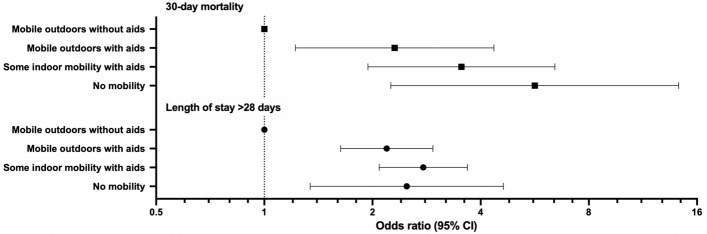
Results of multivariate regression showing odds ratios (with 95% confidence intervals) for mortality at 30-days post-surgery and length of stay in hospital following surgery of > 28 days for different pre-fracture mobility levels compared with patients who were mobile outdoors without aids prior to hip fracture

Patients requiring mobility aids were more likely to have LOS > 28 days compared to patients who were mobile without aids (OR 2.8, 95% CI 2.2–3.6; *p* < 0.001). In the adjusted analysis, the OR for LOS > 28 days was 2.2 (95% CI 1.6–3.0, *p* < 0.001) for patients who were mobile outdoors with aids, 2.8 (95% CI 2.1–3.7, *p* < 0.001) for patients mobile indoors with aids, and 2.5 (95% CI 1.3–4.6, *p* = 0.004) for patients who were not mobile, when compared to the referent group. The adjusted odds ratios for prolonged LOS and 30-day mortality are shown in Fig. 2.

### Post-operative location

After excluding patients who died within 30 days of surgery, worse mobility was associated with a reduced likelihood of returning to the patient’s own home or sheltered housing within 30-days post-operatively. In patients requiring aids the OR for not returning home within 30-days post-operatively was 3.3 (95% CI 2.6–4.2, *p* < 0.001) when compared with the referent group.

In the adjusted analysis, OR for being in a location other than home or sheltered housing at 30 days were 2.1 (95% CI 1.6–2.8, *p* < 0.001) and 4.9 (95% CI 3.6–6.7, *p* < 0.001) for patients who were mobile outdoors using aids, and mobile indoors with aids, respectively, when compared with the referent group.  

A separate regression analysis including patients who died within 30 days of surgery was completed for comparison. Results between the two analyses did not significantly differ. The results of both analyses can be found in Supplementary Tables 5 and 6.

### Post-operative medical complications

Medical complications within 30 days were significantly increased in the groups who were mobile outdoors with aids, 2.0 (95% CI 1.5–2.5) and mobile indoors with aids, 1.8 (95% CI 1.4–2.2). Both groups also showed significantly increased incidence of renal complications and infection within 30-days post-operatively (*p* ≤ 0.001).

**Fig. 3:**
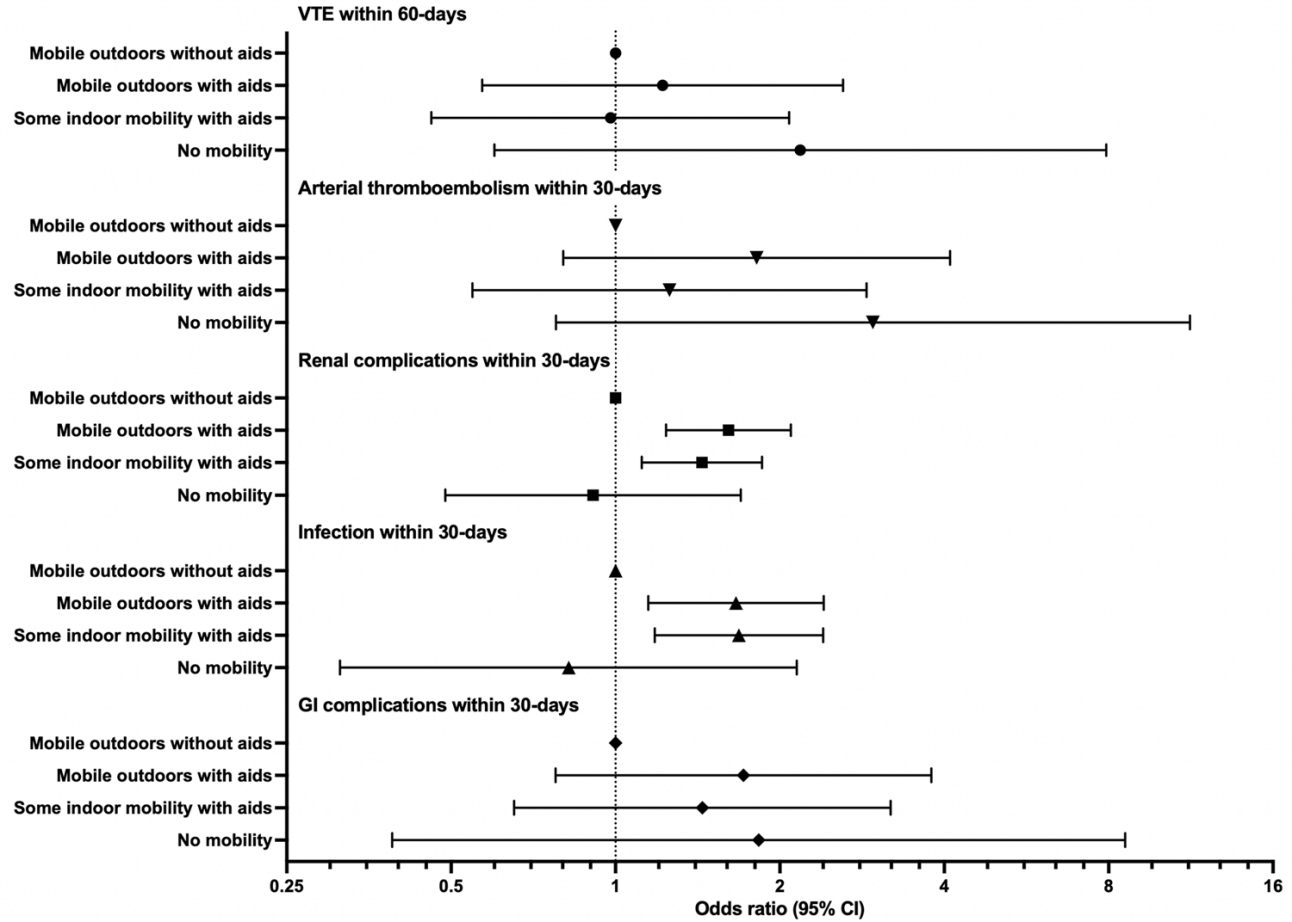
Results of multivariate regression showing odds ratios (with 95% confidence intervals) for post-operative complications for different pre-fracture mobility levels compared with patients who were mobile outdoors without aids prior to hip fracture

In the analysis adjusting for age and sex, worse pre-fracture mobility was associated with an increased likelihood (OR 1.6, 95% CI 1.3–2.0, *p* < 0.001) of suffering complications within 30 days post-operatively compared with the referent group. OR for renal complications within 30 days and infection within 30 days were 1.5 (95% CI 1.2–1.9, *p* = 0.001) and 1.6 (95% CI 1.2–2.3, *p* = 0.004), respectively, for patients requiring aids, compared with the referent group. The adjusted odds ratios for post-operative complications are shown in Fig. 3. Additional univariate association data are available in Supplementary Table 4. The full results of both univariate and adjusted (multivariate) analyses are shown in Supplementary Tables 5 and 6.

The full results of both univariate and adjusted (multivariate) analyses are shown in Supplementary Tables 5 and 6.

### Discrimination analysis

Nine patients had missing NHFS data leaving 1910 patients available for analysis.

For the NHFS, *c*-statistics were 0.681 (0.633–0.729), 0.598 (0.573–0.624) and 0.755 (0.733–0.777) for mortality, post-operative complications, and location other than own home at 30 days post-operatively. *C*-statistics were 0.584 (0.557–0.611) for LOS ≥ 28 days.

For the variable containing NHFS and mobility, c-statistics were 0.696 (0.650–0.742), 0.591 (0.566–0.617) and 0.808 (0.789–0.828) for mortality, post-operative complications, and location other than own home at 30 days post-operatively. *C*-statistics were 0.616 (0.590–0.643) for LOS ≥ 28 days. Results of this analysis are found in Table 2 and Supplementary Fig. 1.

**Table 2:** *C*-statistics (with 95% CI) for post-operative outcomes for NHFS with, and without, pre-fracture mobility (*n* = 1910)

	NHFS	NHFS + Pre-fracture mobility
Mortality at 30 days	0.681 [0.633–0.729]	0.696 [0.65–0.742]
Post-operative complications (excl. PE/DVT) at 30 days	0.598 [0.573–0.624]	0.591 [0.566–0.617]
At location other than own home at 30 days	0.755 [0.733–0.777]	0.808 [0.789–0.828]
Length of stay in hospital ≥ 28 days	0.584 [0.557–0.611]	0.616 [0.59–0.643]

## Discussion

Using prospectively collected data, we demonstrate that worse mobility was independently associated with a range of adverse post-operative including increased post-operative mortality, complications, LOS and an increased likelihood of being discharged to a care home post-operatively.

Poor mobility is recognised to be an adverse prognostic sign in clinical practice, however, very few studies have assessed the association between pre-fracture mobility and outcomes after hip fracture. Mortality has previously been associated with an aggregate score of pre-fracture mobility and NHFS [13]. This study also found that worse pre-fracture mobility was associated with higher mortality, and a modified NHFS with pre-fracture mobility has good discrimination for 30-day mortality [13].

Hjelholt et al. associated pre-fracture mobility, as measured by the Cumulative Ambulation Score (CAS), with mortality at 1-year following fracture [14]. This study also associated mortality with other potential measures of frailty, such as the Charlson Comorbidity Index and Hospital Frailty Score. However, it did not look at other post-operative outcomes. To our knowledge, no other studies have assessed pre-fracture mobility and mortality.

A 2006 study used an assessment of early post-operative mobility to predict post-operative outcomes. The authors reported that worse mobility in the early post-operative period was associated with higher mortality at 1-month, longer post-operative stays and higher incidence of post-operative complications. This study also used a specific cut-off point for the CAS, as a measure of post-operative mobility in the first 3 post-operative days [15].

Another study attempted to predict outcomes following hip fracture surgery using mobility as a variable. Nijmeijer et al. used a measure of pre-fracture mobility (Parker Mobility Score) within a prognostic score for predicting 30-day mortality following hip fracture surgery [16]. This study created the Almelo Hip Fracture Score (AHFS), which was compared with an altered version of the NHFS with regards to discriminating power for 30-day mortality. Aside from using a version of the NHFS with a different measure of cognitive frailty, there were some key differences between our studies. Nijmeijer et al. excluded patients with an indication for total hip replacement, which likely excluded patients who had better functional status and, therefore, better outcomes following surgery [17]. Nijmeijer et al. also excluded patients who had periprosthetic or pathological fractures, which also likely led their cohort to be less complex and at lower risk [16, 18, 19]. This study demonstrated that the AHFS had a greater discriminating power for 30-day mortality than the altered NHFS (AUC 0.82 vs 0.72 respectively); this, therefore, supports our findings that a measure of pre-fracture mobility can improve outcome prediction following hip fracture surgery [16]. They did not measure outcome variables other than 30-day mortality, unlike our study.

The variables of the NHFS were designed so that they were objective and known at admission, making it easier to measure and implement [20]. Despite outperforming the altered NHFS, the AHFS lacks this feature [16].

Neither the NHFS nor our modified score includes perioperative factors. Whilst these factors, such as pre-operative waiting time, type of femoral implant, prolonged operative time and perioperative anaemia [21–23], are known to influence post-operative outcomes, they are by definition not known at the point of admission to hospital. This is where risk scores such as the NHFS have most clinical utility in informing discussions with the patients regarding outcomes.

Discharging patients to their own homes following hip fracture has been a Key Performance Indicator in the NHFD since 2018 [24]. Returning home implies a better recovery to a level not requiring higher level care [25]. In this study a significant proportion of patients were in rehabilitation units at 30-day post-surgery. The proportion increases in groups with lower mobility, other than no mobility, which likely reflects extended rehabilitation for patients requiring walking aids, but poor rehabilitation potential for patients with the lowest level of pre-fracture mobility.

A retrospective analysis by Salar et al. found that, similar to our results, being able to walk independently outdoors was an independent predictor of direct hospital-to-home discharge [26]. This was supported by another study, which found that likelihood of home discharge following hip fracture was correlated with higher CAS [15]. A study from the Irish Hip Fracture Database also found that being independently mobile prior to fracture and early post-operative mobilisation increased likelihood of hospital-to-home discharge [27]. Furthermore, it was reported that early mobilisation of patients with poor pre-fracture mobility lead to a lower in-hospital mortality [28].

For our post-operative location regression analysis, patients were excluded if they died within 30 days of surgery. A further regression analysis which included patients who died within 30 days via a composite outcome did not change our results significantly.

Another UK study identified that different clinical commissioning groups (CCGs) vary in ability to discharge patients directly back home. In some CCGs, who prioritise direct-home-discharge, patients discharged home had longer post-operative stays in hospital than patients discharged to institutional care [29]. This reflects a U-shaped relationship between LOS and measures associated with physical or cognitive frailty. This is likely as poor mobility or cognition can necessitate discharge to care homes, which can provide a greater level of care than can be delivered in a patient’s home, and thus faster discharge at a lower level of function is feasible. Lower risk patients are more likely to have higher levels of independence prior to injury; often increasing their LOS to access physiotherapy and occupational therapy to improve the chance of returning to pre-fracture levels of independence. This rehabilitation is at the expense of a longer LOS. We previously reported a U-shaped distribution of LOS with respect to the NHFS [5]. The pre-existing characteristics influencing recovery and rehabilitation potential following surgery are closely related to pre-fracture residence, as they are both indicators of independence. The relationship between pre-fracture residence and LOS has not been analysed and is, therefore, a limitation in this study.

Although there are limited data on the impact of pre-fracture mobility on discharge destination, other studies have examined the effect of risk scores on discharge destination. Moppett et al. used the NHFS, to discriminate between patients who returned home following hip fracture, and those who did not [30].

In our study, pre-fracture mobility was associated with all complication rates within 30 days of surgery with and without adjustments for age and sex. When looking at specific complications, only renal complications, and infections within 30 days of surgery were associated with pre-fracture mobility. We hypothesised that the higher complication rates in groups with worse mobility were due to the presence of the frailty syndrome. Frailty would increase the number of post-operative complications because patients living with frailty are less able to respond to physiological stressors; complications would also be expected to be more severe. It is recognised that prolonged hospitalisation and bed rest are risk factors for respiratory infections [31]. Our complications data did not contain measures of severity, therefore future work could incorporate measures of severity. No other studies have analysed the relationship between pre-fracture mobility and post-operative complications.

Our previous study demonstrated the NHFS to have good discrimination for mortality, post-operative outcomes, location and length of stay [5]. The analysis in this study used pre-fracture mobility as a measure of frailty in combination with the NHFS to demonstrate that the discrimination of the NHFS can be improved by including measures of frailty. Frailty has been found to be independently associated with increased mortality, morbidity, and length of stay in hip fracture patients [6–9]. The addition of pre-fracture mobility moderately improved the power of the NHFS for discriminating post-operative location and length of stay in this study. It did not significantly change the *c*-statistics for 30-day mortality and did not improve discrimination of post-operative complications. We have previously reported that the NHFS alone is a poor discriminator of post-operative complications [5].

Our study had several strengths, we have independently associated a breadth of post-operative outcomes with pre-fracture mobility, many of which have not previously been reported in hip fracture. Our study used prospectively collected data from a single trauma unit in Northumberland between 2014 and 2019 and contains a larger sample size than most hip fracture studies. This sample size facilitated comprehensive univariate and multivariate regression analyses of the variables assessed.

The demographics of our cohort were largely representative of the national hip fracture cohort, meaning results should be transferable to the wider hip fracture population. Pre-fracture mobility is routinely collected as part of NHFD data; therefore, other centres could use this to replicate our analyses in their cohorts, and in future we may seek to collaborate with other centres to enable larger, multi-centre analyses.

Limitations of our study included using HES data, which do not include an exhaustive list of post-operative medical complications. HES data are collected from hospital documentation and contains clinical, patient characteristic and administrative data. Its reliability is dependent on the quality of documentation and input of data by coders and, therefore, may under or over report complications [32, 33]. Variation in the reliability of HES data may go some way in explaining the discordance between the enhanced prediction of LOS and discharge location, but lack of improvement in prediction of complications. Furthermore, post-operative complications are relatively uncommon, and the group with the smallest sample size (no mobility) are likely to have a higher prevalence of conditions that can preclude accurate and timely diagnosis of complications (such as severe dementia and multimorbidity). The large overall sample size of our cohort is likely to ameliorate some of these effects.

Post-surgical complications DVT and PE were prospectively collected within 60 days of surgery, whereas other complications were measured within 30 days of surgery. This meant they could not be analysed together with other post-surgical complication data for a more accurate measure of total complication rates, which was also measured within 30 days of surgery. Future studies could consider the use of additional or alternative data sources for determining complication rates.

Our study excluded patients who did not undergo operative management so the results cannot be assumed to extend to those who did not undergo surgery, although this is a very small proportion of hip fractures. Despite our cohort size, our study includes patients from only a single trust in North-East England an area which has a deprivation index higher than the national average. Deprivation has been associated with a higher mortality in hip fracture patients and our results may not necessarily translate to areas with a lower deprivation index [34–36].

As different CCGs can vary in LOS due to prioritising direct-to-home discharge, using a single centre could be a disadvantage when applying results to other centres [29].

In conclusion, patients with better mobility, such as patients who were mobile without aids, had significantly improved outcomes than those with worse mobility. The mobility variable was able to independently predict outcomes and enhanced risk prediction when combined with the NHFS. Scores such as the NHFS can be improved by the addition of such a variable to predict 30-day mortality, LOS and discharge location more accurately. This information, which is available at admission, could facilitate prognostication, discharge planning, bed management and risk aversion, as well as informing discussions between clinical teams, patients and their relatives about post-operative status. Furthermore, we demonstrate the potential of analysing prospectively collected datasets in clinical practice; allowing for the study of populations that are not easily consented into traditional observational studies.

## Supplementary Information

Below is the link to the electronic supplementary material.

Supplementary file1 (DOCX 198 KB)

## Data Availability

Any data queries can be directed to the corresponding author by email.
