# Supplementary material for: Using pre-fracture mobility to augment prediction of post-operative outcomes in hip fracture
Source: Eur Geriatr Med. 2023 Mar 31;14(2):285–93. doi: 10.1007/s41999-023-00767-0 (PMC10113355; doi:10.1007/s41999-023-00767-0)
Supplement: Supplementary file 1 — Supplementary file1 (DOCX 198 KB) [file 41999_2023_767_MOESM1_ESM.docx]

**Using pre-fracture mobility to augment prediction of post-operative outcomes after hip fracture surgery: Supplementary material**

Thomas A Stubbs^1^, William J Doherty^1^, Andrew Chaplin^2^, Sarah Langford^2^, Mike R Reed^2^, Avan A Sayer^1^, Miles D Witham*^1^, Antony K Sorial*^2,3^

^1^ AGE Research Group, NIHR Newcastle Biomedical Research Centre, Translational and Clinical Research Institute, Faculty of Medical Sciences, Newcastle University and Newcastle Upon Tyne Hospitals NHS Foundation Trust, Newcastle-upon-Tyne, NE4 5PL, UK.

^2^ Department of Trauma and Orthopaedics, Northumbria Healthcare NHS Foundation Trust, Newcastle upon Tyne, NE27 0QJ, UK.

^3^ Institute for Cell and Molecular Biosciences, Newcastle University, International Centre for Life, Newcastle upon Tyne, NE1 3BZ, UK.

**Supplementary Table 1.** Study variables and their data sources.

**Supplementary Table 2.** ICD-10 or OPCS-4 codes of measured post-operative complications.

**Supplementary Table 3.** Baseline characteristics of all included patients (n=1919) and patients admitted from their own homes or sheltered housing (n=1333).

**Supplementary Table 4.** Univariate association of pre-fracture mobility with post-operative outcomes.

**Supplementary Table 5.** Univariate regression showing odds ratios (with 95% confidence intervals) for post-operative outcomes for different levels of pre-fracture mobility.

**Supplementary Table 6.** Multivariate regression adjusting for age and sex, showing odds ratios (with 95% confidence intervals) for post-operative outcomes for different levels of pre-fracture mobility.

**Supplementary Fig. 1** Receiver Operating Characteristic (ROC) curves for the NHFS and NHFS with mobility with reference line. Curves shown are for 30-day mortality (a), post-operative complications (excl. PE/DVT) at 30-days (b), location other than own home at 30-days (c) and Length of stay in hospital ≥28 days (d).

| **Variables** | **Data source** |
| --- | --- |
| Demographics | |
| Age | Routinely documented by medical team and NHFD nurses on admission |
| Sex |  |
| Pre-fracture mobility |  |
| NHFS | Calculated by orthopaedic registars |
| Operative characteristics | |
| Date of surgery | Documented by medical professionals |
| Post-operative outcomes | |
| Residence at 30- and 120-days | Collected by senior specialist NHFD nurse via telephone interview or documentation by medical professionals on ward |
| Mobility at 30- and 120-days |  |
| Mortality at 30- and 120-days |  |
| Complications at 30- and 60-days | Hospital Episode Statistics (HES) Data |
| Length of stay | Calculated from admission data to discharge date |

**Supplementary Table 1** Study variables and their data sources

| **Post-operative complications** | **ICD-10 or OPSC-4 code** |
| --- | --- |
| DVT in 60 days | ICD10 I801, I802, I803, I808, I809 |
| PE in 60 days | ICD10 I26 |
| Stroke within 30 days | ICD10 I60-I64 |
| TIA within 30 days | ICD10 G45 |
| GI bleed within 30 days | ICD10 K920, K922 |
| Renal failure within 30 days | ICD10 N17 |
| Urinary retention within 30 days | ICD10 R33 |
| UTI within 30 days | ICD10 N390 |
| MI within 30 Days | ICD10 I21, I22 |
| Pneumonia within 30 days | ICD10 J18, J22 plus Y83 |
| Thrombocytopenia within 30 days | ICD10 D696 or D695 plus Y442 |
| Ileus/intestinal blockage within 30 days | ICD10 K56, K913 |
| Blood transfusion within stay in hospital | OPCS4.4 X33 |

**Supplementary Table 2** ICD-10 or OPCS-4 codes of measured post-operative complications

| **Variables** | All patients (n=1919) | Patients admitted from own homes or sheltered housing (n=1333) |
| --- | --- | --- |
| Age |  |  |
| Mean (SD) | 82.6 (8.2) | 81.6 (8.3) |
| Median (IQR) | 83 (78-88) | 82 (76-88) |
| Female sex, n (%) | 1357 (70.7) | 980 (69.4) |
| Pre-fracture mobility, n (%) |  |  |
| Mobile without aids | 550 (28.7) | 511 (36.2) |
| Mobile outdoors with aids | 565 (29.4) | 522 (36.9) |
| Mobile indoors with aids | 743 (38.7) | 368 (26.0) |
| Not mobile | 61 (3.2) | 12 (0.8) |
| Pre-fracture location, n (%) |  |  |
| Own home/sheltered housing | 1413 (73.6) | - |
| Residential care | 381 (19.9) | - |
| Nursing care | 78 (4.1) | - |
| Hospital | 47 (2.4) | - |
| LOS |  |  |
| Mean (SD) | 24.1 (21.5) | 24.8 (21.9) |
| Median (IQR) | 18 (10-30) | 18 (10-30) |
| NHFS |  |  |
| Mean (SD) | 5.0 (1.6) | 4.5 (1.4) |
| Median (IQR) | 5 (4-6) | 5 (4-5) |
| ASA |  |  |
| Mean (SD) | 2.9 (0.7) | 2.7 (0.7) |

**Supplementary Table 3** Baseline characteristics of all included patients (n=1919) and patients admitted from their own homes or sheltered housing (n=1333)

|  | **Mobile outdoors without aids (n=550)** | **Mobile outdoors with aids (n=565)** | **Mobile indoors with aids (n=743)** | **No mobility (n=61)** | **P-value** |
| --- | --- | --- | --- | --- | --- |
| Length of stay in days (n, %) |  |  |  |  |  |
| <7 | 143 (26.7) | 52 (9.8) | 85 (12.6) | 9 (17.0) | **<0.001**^a^ |
| 7-13 | 145 (27.1) | 109 (20.6) | 112 (16.6) | 9 (17.0) |  |
| 14-20 | 106 (19.8) | 111 (21.0) | 106 (15.7) | 10 (18.9) |  |
| 21-27 | 58 (10.8) | 91 (17.2) | 127 (18.8) | 7 (13.2) |  |
| >28 | 84 (15.7) | 166 (31.4) | 244 (36.2) | 18 (34.0) |  |
| Mean (SD) | 18.1 (17.5) | 26.2 (21.7) | 28.5 (23.7) | 26.6 (22.9) | **<0.001**^c^ |
| Median (IQR) | 13 (7-21) | 20 (11-31) | 21 (11-36) | 19 (11-35) |  |
| Residence at 30-days* (n, %) |  |  |  |  |  |
| Own home or sheltered housing | 385 (77.2) | 288 (58.5) | 137 (41.3) | 7 (70.0) | **<0.001**^a^ |
| Residential care | 13 (2.6) | 20 (4.1) | 23 (6.9) | 0 (0.0) |  |
| Nursing care | 1 (0.2) | 3 (0.6) | 5 (1.5) | 0 (0.0) |  |
| Rehabilitation unit | 93 (18.6) | 161 (32.7) | 164 (49.4) | 3 (30.0) |  |
| Hospital | 7 (1.4) | 20 (4.1) | 13 (3.9) | 0 (0.0) |  |
| Mortality at 30-days (n, %) |  |  |  |  |  |
| Deceased | 14 (2.5) | 36 (6.4) | 69 (9.3) | 8 (13.1) | **<0.001**^a^ |
| Post-operative complications (n, %) |  |  |  |  |  |
| Stroke within 30 days | 4 (0.7) | 8 (1.4) | 9 (1.2) | 3 (4.9) | 0.08^b^ |
| Transient ischaemic attack within 30 days | 3 (0.5) | 0 (0) | 1 (0.1) | 0 (0) | 0. 25^b^ |
| GI bleed within 30 days | 5 (0.9) | 13 (2.3) | 14 (1.9) | 1 (1.6) | 0.27^b^ |
| Renal failure within 30 days | 60 (10.9) | 85 (15.0) | 108 (14.5) | 3 (4.9) | **0.031**^a^ |
| Urinary retention within 30 days | 65 (11.8) | 97 (17.2) | 120 (16.2) | 10 (16.4) | 0.07^a^ |
| UTI within 30 days | 41 (7.5) | 77 (13.6) | 94 (12.7) | 5 (8.2) | **0.005**^a^ |
| MI within 30 days | 5 (0.9) | 10 (1.8) | 8 (1.1) | 0 (0) | 0.57^b^ |
| Pneumonia within 30 days | 11 (2.0) | 13 (2.3) | 31 (4.2) | 0 (0) | 0.05^b^ |
| Thrombocytopenia within 30 days | 5 (0.9) | 11 (1.9) | 8 (1.1) | 1 (1.6) | 0.35^b^ |
| Ileus or intestinal blockage within 30 days | 5 (0.9) | 7 (1.2) | 7 (0.9) | 1 (1.6) | 0.72^b^ |
| Any complication (excl. DVT/ PE/ transfusion) within 30 days | 157 (28.5) | 248 (43.9) | 307 (41.3) | 19 (31.1) | **<0.001**^a^ |
| Deep vein thrombosis within 60 days | 6 (1.1) | 8 (1.4) | 10 (1.3) | 1 (1.6) | 0.85^b^ |
| Pulmonary embolism within 60 days | 7 (1.3) | 7 (1.2) | 6 (0.8) | 2 (3.3) | 0.27^b^ |
| Blood transfusion during stay in hospital | 11 (2.0) | 14 (2.5) | 15 (2.0) | 0 (0) | 0.78^b^ |

**Supplementary Table 4** Univariate association of pre-fracture mobility with post-operative outcomes. Significant results highlighted in bold. N = 1919. a = Chi-square test; b = Fisher's exact test; c = One-way ANOVA.

*Analysis of residence at 30-days only included patients admitted from their own homes or sheltered housing

|  | **a) Mortality at 30-days (n=1919)** | | **b) Length of stay >28 days (n=1785)** | |
| --- | --- | --- | --- | --- |
| **Pre-fracture mobility** | OR [95% CI] | P-value | OR [95% CI] | P-value |
| **Mobile outdoors without aids** | Reference | - | Reference | - |
| **Mobile outdoors with aids** | 2.61 [1.39 - 4.89] | **0.003** | 2.44 [1.82 - 3.27] | **<0.001** |
| **Mobile indoors with aids** | 3.92 [2.18 - 7.04] | **<0.001** | 3.14 [2.38 - 4.13] | **<0.001** |
| **No mobility** | 5.78 [2.32 - 14.41] | **<0.001** | 2.58 [1.4 - 4.77] | **0.002** |
| **Non-mobile without aids total** | 3.44 [1.96 - 6.06] | **<0.001** | 2.80 [2.17 - 3.62] | **<0.001** |

|  | **c) Location at 30-days post surgery excluding patients who died within 30-days (n=1333)** | | | | | | | | | | | |
| --- | --- | --- | --- | --- | --- | --- | --- | --- | --- | --- | --- | --- |
| **Pre-fracture mobility** | **Own home** | | **Residential care** | | **Nursing care** | | **Rehabilitation unit** | | **Hospital** | | **Location other than home** | |
|  | OR [95% CI] | P-value | OR [95% CI] | P-value | OR [95% CI] | P-value | OR [95% CI] | P-value | OR [95% CI] | P-value | OR [95% CI] | P-value |
| **Mobile outdoors without aids** | Reference | - | Reference | - | Reference | - | Reference | - | Reference | - | Reference | - |
| **Mobile outdoors with aids** | 0.42 [0.32 - 0.55] | **<0.001** | 1.58 [0.78 - 3.22] | 0.204 | 3.06 [0.32 - 29.5] | 0.334 | 2.12 [1.58 - 2.85] | **<0.001** | 2.98 [1.25 - 7.11] | **0.014** | 2.39 [1.82 - 3.15] | **<0.001** |
| **Mobile indoors with aids** | 0.18 [0.14 - 0.25] | **<0.001** | 2.78 [1.39 - 5.58] | **0.004** | 7.615 [0.89 - 65.5] | 0.064 | 4.26 [3.12 - 5.82] | **<0.001** | 2.86 [1.13 - 7.26] | **0.027** | 5.45 [4.02 - 7.39] | **<0.001** |
| **No mobility** | 0.69 [0.18 - 2.72] | 0.596 | - | . | - | . | 1.87 [0.48 - 7.37] | 0.371 | - | . | 1.45 [0.37 - 5.69] | 0.596 |
| **Non-mobile without aids total** | 0.30 [0.24 - 0.39] | **<0.001** | 2.03 [1.08 - 3.82] | **0.028** | 4.82 [0.60 - 38.7] | 0.139 | 2.83 [2.17 - 3.69] | **<0.001** | 2.90 [1.27 - 6.60] | **0.011** | 3.30 [2.57 - 4.23] | **<0.001** |

|  | **d) Location at 30-days post surgery (n=1413)** | | | | | | | | | | | |
| --- | --- | --- | --- | --- | --- | --- | --- | --- | --- | --- | --- | --- |
| **Pre-fracture mobility** | **Own home** | | **Residential care** | | **Nursing care** | | **Rehabilitation unit** | | **Hospital** | | **Location other than home** | |
|  | OR [95% CI] | P-value | OR [95% CI] | P-value | OR [95% CI] | P-value | OR [95% CI] | P-value | OR [95% CI] | P-value | OR [95% CI] | P-value |
| **Mobile outdoors without aids** | Reference | - | Reference | - | Reference | - | Reference | - | Reference | - | Reference | - |
| **Mobile outdoors with aids** | 0.40 [0.31 - 0.53] | **<0.001** | 1.53 [0.75 - 3.10] | 0.243 | 2.95 [0.31 - 28.4] | 0.350 | 2.01 [1.50 - 2.68] | **<0.001** | 2.87 [1.20 - 6.84] | **0.018** | 2.48 [1.91 - 3.23] | **<0.001** |
| **Mobile indoors with aids** | 0.17 [0.13 - 0.23] | **<0.001** | 2.55 [1.28 - 5.11] | **0.008** | 7.03 [0.82 - 60.3] | 0.076 | 3.61 [2.67 - 4.90] | **<0.001** | 2.64 [1.04 - 6.68] | **0.041** | 5.80 [4.32 - 7.78] | **<0.001** |
| **No mobility** | 0.46 [0.14 - 1.47] | 0.189 | - | . | - | . | 1.50  [0.40 - 5.64] | 0.550 | - | . | 2.18 [0.68 - 7.00] | 0.189 |
| **Non-mobile without aids total** | 0.29 [0.23 - 0.37] | **<0.001** | 1.92 [1.02 - 3.6] | **0.043** | 4.56 [0.57 - 36.6] | 0.153 | 2.83 [2.17 - 3.69] | **<0.001** | 2.73 [1.20 - 6.22] | **0.017** | 3.48 [2.73 - 4.42] | **<0.001** |

|  | **e) Post-operative complications (n=1919)** | | | | | | | | | | | |
| --- | --- | --- | --- | --- | --- | --- | --- | --- | --- | --- | --- | --- |
| **Pre-fracture mobility** | **VTE event within 60-days** | | **Arterial TE event within 30-days** | | **Renal complication within 30-days** | | **Infection within 30-days** | | **GI complication within 30-days** | | **Any complication within 30-days** | |
|  | OR [95% CI] | P-value | OR [95% CI] | P-value | OR [95% CI] | P-value | OR [95% CI] | P-value | OR [95% CI] | P-value | OR [95% CI] | P-value |
| **Mobile outdoors without aids** | Reference | - | Reference | - | Reference | - | Reference | - | Reference | - | Reference | - |
| **Mobile outdoors with aids** | 1.13 [0.53 - 2.39] | 0.756 | 1.98 [0.88 - 4.44] | 0.098 | 1.83 [1.42 - 2.37] | **<0.001** | 1.85 [1.29 - 2.67] | **0.001** | 1.78 [0.81 - 3.89] | 0.15 | 1.96 [1.53 - 2.51] | **<0.001** |
| **Mobile indoors with aids** | 0.91 [0.43 - 1.91] | 0.801 | 1.41 [0.62 - 3.18] | 0.411 | 1.68 [1.31 - 2.14] | **<0.001** | 1.96 [1.39 - 2.77] | **<0.001** | 1.42 [0.65 - 3.07] | 0.377 | 1.76 [1.39 - 2.23] | **<0.001** |
| **No mobility** | 2.14 [0.59 - 7.72] | 0.247 | 3.11 [0.82 - 11.81] | 0.096 | 0.97 [0.53 - 1.80] | 0.932 | 0.87 [0.34 - 2.28] | 0.782 | 1.83 [0.39 - 8.55] | 0.442 | 1.13 [0.64 - 2.01] | 0.67 |
| **Non-mobile without aids total** | 1.05 [0.55 - 2.01] | 0.878 | 1.72 [0.82 - 3.57] | 0.149 | 1.70 [1.36 - 2.13] | **<0.001** | 1.86 [1.35 - 2.57] | **<0.001** | 1.58 [0.79 - 3.20] | 0.199 | 1.81 [1.46 - 2.24] | **<0.001** |

**Supplementary Table 5** Results of univariate regression showing odds ratios (with 95% confidence intervals) for post-operative outcomes for different levels of pre-fracture mobility, compared to patients who were mobile without aids prior to hip fracture. Significant results highlighted in bold

|  | **a) Mortality at 30-days (n=1919)** | | **b) Length of stay >28 days (n=1785)** | |
| --- | --- | --- | --- | --- |
| **Pre-fracture mobility** | OR [95% CI] | P-value | OR [95% CI] | P-value |
| **Mobile outdoors without aids** | Reference | - | Reference | - |
| **Mobile outdoors with aids** | 2.31 [1.22 - 4.36] | **0.01** | 2.19 [1.63 - 2.95] | **<0.001** |
| **Mobile indoors with aids** | 3.54 [1.94 - 6.44] | **<0.001** | 2.77 [2.09 - 3.67] | **<0.001** |
| **No mobility** | 5.66 [2.25 - 14.23] | **<0.001** | 2.49 [1.342- 4.63] | **0.004** |
| **Non-mobile without aids total** | 3.08 [1.74 - 5.47] | **<0.001** | 2.50 [1.92 - 3.24] | **<0.001** |

|  | **c) Location at 30-days post surgery, excluding patients who died within 30-days (n=1333)** | | | | | | | | | | | |
| --- | --- | --- | --- | --- | --- | --- | --- | --- | --- | --- | --- | --- |
| **Pre-fracture mobility** | **Own home** | | **Residential care** | | **Nursing care** | | **Rehabilitation unit** | | **Hospital** | | **Location other than home** | |
|  | OR [95% CI] | P-value | OR [95% CI] | P-value | OR [95% CI] | P-value | OR [95% CI] | P-value | OR [95% CI] | P-value | OR [95% CI] | P-value |
| **Mobile outdoors without aids** | Reference | - | Reference | - | Reference | - | Reference | - | Reference | - | Reference | - |
| **Mobile outdoors with aids** | 0.48 [0.36 - 0.63] | **<0.001** | 1.29 [0.63 - 2.64] | 0.488 | 3.35 [0.34 - 33.1] | 0.301 | 1.85 [1.37 - 2.50] | **<0.001** | 3.44 [1.42 - 8.31] | 0.006 | 2.1 [1.59 - 2.78] | **<0.001** |
| **Mobile indoors with aids** | 0.21 [0.15 - 0.28] | **<0.001** | 2.16 [1.07 - 4.38] | **0.032** | 7.78 [0.88 - 69.1] | 0.066 | 3.77 [2.74 - 5.19] | **<0.001** | 3.60 [1.40 - 9.28] | **0.008** | 4.88 [3.58 - 6.67] | **<0.001** |
| **No mobility** | 0.51 [0.13 - 2.11] | 0.355 | - | . | - | . | 2.57 [0.62 - 10.6] | 0.193 | - | . | 1.95 [0.47 - 7.98] | 0.355 |
| **Non-mobile without aids total** | 0.34 [0.27 - 0.44] | **<0.001** | 1.63 [0.86 - 3.08] | 0.135 | 5.06 [0.62 - 41.6] | 0.132 | 2.49 [1.90 - 3.26] | **<0.001** | 3.42 [1.48 - 7.88] | **0.004** | 2.92 [2.27 - 3.77] | **<0.001** |

|  | **d) Location at 30-days post surgery (n=1413)** | | | | | | | | | | | |
| --- | --- | --- | --- | --- | --- | --- | --- | --- | --- | --- | --- | --- |
| **Pre-fracture mobility** | **Own home** | | **Residential care** | | **Nursing care** | | **Rehabilitation unit** | | **Hospital** | | **Location other than home** | |
|  | OR [95% CI] | P-value | OR [95% CI] | P-value | OR [95% CI] | P-value | OR [95% CI] | P-value | OR [95% CI] | P-value | OR [95% CI] | P-value |
| **Mobile outdoors without aids** | Reference | - | Reference | - | Reference | - | Reference | - | Reference | - | Reference | - |
| **Mobile outdoors with aids** | 0.46 [0.35 - 0.60] | **<0.001** | 1.25 [0.61 - 2.56] | 0.539 | 3.326 [0.33 - 32.3] | 0.311 | 1.77 [1.32 - 2.39] | **<0.001** | 3.33 [1.38 - 8.05] | 0.007 | 2.18 [1.66 - 2.86] | **<0.001** |
| **Mobile indoors with aids** | 0.19 [0.14 - 0.26] | **<0.001** | 1.98 [0.98 - 4.00] | 0.058 | 7.33 [0.82 - 65.3] | 0.074 | 3.16 [2.32 -4.32] | **<0.001** | 3.32 [1.29 - 8.55] | **0.013** | 5.21 [3.85 - 7.04] | **<0.001** |
| **No mobility** | 0.33 [0.10 - 1.09] | 0.068 | - | . | - | . | 2.02 [0.52 - 7.85] | 0.313 | - | . | 3.08 [0.92 - 10.29] | 0.068 |
| **Non-mobile without aids total** | 0.32 [0.25 - 0.41] | **<0.001** | 1.54 [0.81 - 2.92] | 0.185 | 4.83 [0.59 - 39.8] | 0.143 | 2.27 [1.74 - 2.96] | **<0.001** | 3.24 [1.40 - 7.48] | **0.006** | 3.09 [2.42 - 3.95] | **<0.001** |

|  | **e) Post-operative complications (n=1919)** | | | | | | | | | | | |
| --- | --- | --- | --- | --- | --- | --- | --- | --- | --- | --- | --- | --- |
| **Pre-fracture mobility** | **VTE event within 60-days** | | **Arterial TE event within 30-days** | | **Renal complication within 30-days** | | **Infection within 30-days** | | **GI complication within 30-days** | | **Any complication within 30-days** | |
|  | OR [95% CI] | P-value | OR [95% CI] | P-value | OR [95% CI] | P-value | OR [95% CI] | P-value | OR [95% CI] | P-value | OR [95% CI] | P-value |
| **Mobile outdoors without aids** | Reference | - | Reference | - | Reference | - | Reference | - | Reference | - | Reference | - |
| **Mobile outdoors with aids** | 1.22 [0.57 - 2.61] | 0.613 | 1.81 [0.80 - 4.10] | 0.153 | 1.61 [1.24 - 2.10] | **<0.001** | 1.66 [1.15 - 2.41] | **0.007** | 1.72 [0.78 - 3.79] | 0.182 | 1.74 [1.35 - 2.25] | **<0.001** |
| **Mobile indoors with aids** | 0.98 [0.46 - 2.08] | 0.95 | 1.26 [0.55 - 2.88] | 0.59 | 1.44 [1.12 - 1.86] | **0.005** | 1.68 [1.18 - 2.40] | **0.004** | 1.44 [0.65 - 3.19] | 0.366 | 1.55 [1.21 - 1.98] | **<0.001** |
| **No mobility** | 2.18 [0.60 - 7.92] | 0.235 | 2.96 [0.78 - 11.27] | 0.111 | 0.91 [0.49 - 1.70] | 0.766 | 0.82 [0.31 - 2.15] | 0.687 | 1.83 [0.39 - 8.58] | 0.444 | 1.07 [0.60 - 1.91] | 0.819 |
| **Non-mobile without aids total** | 1.13 [0.59 - 2.20] | 0.71 | 1.28  [0.60 - 2.76] | 0.528 | 1.48 [1.18 - 1.86] | **0.001** | 1.63 [1.17 - 2.26] | **0.004** | 1.58 [0.77 - 3.24] | 0.21 | 1.60 [1.28 – 2.00] | **<0.001** |

**Supplementary Table 6** Results of multivariate regression adjusting for age and sex, showing odds ratios (with 95% confidence intervals) for post-operative outcomes for different levels of pre-fracture mobility, compared to patients who were mobile without aids prior to hip fracture. Significant results highlighted in bold

**Supplementary Fig. 1** Receiver Operating Characteristic (ROC) curves for the NHFS (Blue) and NHFS + mobility (Green) with reference line (Red). Curves shown are for 30-day mortality (a), post-operative complications (excl. PE/DVT) at 30-days (b), location other than own home at 30-days (c) and Length of stay in hospital ≥28 days (d)
